# Die Effektivität stationärer Behandlung von Psychosen – erste Ergebnisse einer naturalistischen Studie

**DOI:** 10.1007/s00115-025-01871-1

**Published:** 2025-08-19

**Authors:** Lara A. Martini, Alexander H. J. Sahm, Michael Odenwald, Anna Becker, Daniela Mier

**Affiliations:** Klinische Psychologie und Psychotherapie, Universität Konstanz, Konstanz, Deutschland

**Keywords:** Psychose, Stationäre Behandlung, Practice-based evidence, Naturalistische Studie, Einsicht, Psychosis, Inpatient treatment, Practice-based evidence, Naturalistic study, Insight

## Abstract

**Hintergrund:**

Eine wichtige Säule in der Behandlung von Menschen mit psychotischen Störungen ist die stationäre Versorgung. Während sich die Wirksamkeit psychotherapeutischer und pharmakologischer Interventionen für psychotische Störungen auf eine breite wissenschaftliche Evidenz stützt, mangelt es an naturalistischen Studien, die die Effektivität der stationären Behandlung belegen.

**Methodik:**

Bei 65 Patient:innen mit psychotischen Störungen, die auf einer spezialisierten Station nach aktuellen Leitlinien behandelt wurden, erfolgte zu Beginn des Aufenthalts und bei Entlassung eine Erhebung mittels Interviews und Fragebögen zu Symptomatik, Lebensqualität und Funktionsniveau.

**Ergebnisse:**

Der Aufenthalt führt zu einer Verbesserung der Gesamtsymptomatik sowie der positiven und affektiven Symptome, der Desorganisation, des Funktionsniveaus und der Lebensqualität, nicht aber der Negativsymptomatik. Außerdem ließ sich eine Verbesserung der Krankheitseinsicht beobachten. Die Selbsteinschätzung positiver Symptome zeigte zu beiden Zeitpunkten eine Übereinstimmung mit der Fremdeinschätzung.

**Diskussion:**

Es ergab sich eine Verbesserung in allen Symptomkategorien, außer in der Negativsymptomatik. Die Ergebnisse demonstrieren die Effektivität der stationären Therapie nach den Leitlinien zur Schizophreniebehandlung und unterstreichen die Notwendigkeit, Patient:innen mit Psychose auch im stationären Kontext ein umfassendes therapeutisches Angebot zukommen zu lassen.

**Zusatzmaterial online:**

Zusätzliche Informationen sind in der Online-Version dieses Artikels (https://doi.org/10.1007/s00115-025-01871-1) enthalten.

## Abstract (en)

**Background:**

An important pillar in the treatment of patients with psychotic disorders is inpatient care. While the efficacy of psychotherapeutic and pharmacological interventions for psychotic disorders is based on broad scientific evidence, there is a lack of naturalistic studies demonstrating the effectiveness of inpatient treatment.

**Methodology:**

Interviews and questionnaires on symptoms, quality of life, and level of functioning were collected from 65 patients with psychotic disorders who were treated on a specialized ward according to current guidelines at the beginning of the hospital stay and on discharge.

**Results:**

The inpatient stay led to an improvement in overall symptoms as well as positive and affective symptoms, disorganization, level of functioning, and quality of life, but not in negative symptoms. In addition, an improvement of insights into the disorder was observed. The self-rating of positive symptoms was consistent with the observer rating at both time points.

**Discussion:**

Improvements were apparent in all symptom categories, except for negative symptoms. The results demonstrate the effectiveness of inpatient therapy according to current guidelines for the treatment of schizophrenia and underline the necessity of providing patients with psychosis with a comprehensive therapeutic offer also in the inpatient context.

Psychosen sind komplexe psychische Phänomene, die Störungen aus dem schizophrenen Formenkreis kennzeichnen, jedoch auch bei anderen psychischen Störungen, wie der bipolaren Störung oder Substanzkonsumstörungen, auftreten können. Aufgrund der oft akuten Beeinträchtigung und der mangelnden ambulanten psychotherapeutischen Versorgung kommt der stationären Behandlung besondere Bedeutung zu. Da nur wenige Studien aus dem deutschsprachigen Raum zur Effektivität dieses Behandlungssettings vorliegen, werden hier die ersten Ergebnisse einer naturalistischen Studie zur Behandlung auf einer auf Psychosen spezialisierten Therapiestation präsentiert.

## Hintergrund

Psychosen sind durch Realitätsverlust, Wahn, Halluzinationen und formale Denkstörungen charakterisiert. Die 12-Monats-Prävalenz psychotischer Störungen liegt in Deutschland bei 2,6 % [9], dabei gehen Psychosen mit einer deutlichen Einschränkung der gesellschaftlichen Teilhabe einher. Alleine durch die Schizophrenie entstehen in Deutschland jährlich gesellschaftliche Kosten in Höhe von 9,63 bis 13,52 Mrd. € [8]. Aktuelle Leitlinien zur Behandlung der Schizophrenie [6] empfehlen eine Kombination aus Pharmakotherapie und psychotherapeutischen und psychosozialen Interventionen. Dies deckt sich zudem mit internationalen Leitlinien [14]. Entgegen diesen Empfehlungen erhalten Patient:innen mit Psychosen jedoch nur selten ambulante Psychotherapie [30]; gleichzeitig zählt die Schizophrenie zu den häufigsten Störungen, die stationär psychiatrisch versorgt werden [34].

Während allgemein die Wirksamkeit der Behandlung mit pharmakologischen und psychotherapeutischen Interventionen auf Basis zahlreicher Studien und Metaanalysen als gesichert angesehen werden kann [21, 22], ist die Effektivität stationärer Therapien weniger erforscht [24]. Vorliegende metaanalytische Auswertungen untersuchen vor allem die Wirksamkeit kognitiver Verhaltenstherapie (KVT) im stationären Setting im Vergleich zu Standardinterventionen und berichten kleine Effekte auf Positivsymptomatik (Standardisierte Mittelwertdifferenz (SMD) = 0,2–0,39) und begrenzt Effekte auf die Negativsymptomatik (SMD = 0,16–0,25; [1, 37]). Die dabei vorwiegend untersuchten randomisierten kontrollierten Studien lassen sich aufgrund selektiver Rekrutierung und forschungsgeleiteter strikt manualisierter Interventionen aber in der Regel nur begrenzt auf den Alltag der stationären Versorgung generalisieren. Obwohl einige Interventionsstudien im naturalistischen Setting durchgeführt wurden (z. B. [35]), sind Studien mit naturalistischen Interventionen im Sinne einer „practice-based evidence“ zur Untersuchung der Effektivität einer leitliniengerechten Behandlung in der alltäglichen stationären Anwendung sowohl in Deutschland als auch international rar [4]. Beispielsweise konnte in einer Studie in Schweden beobachtet werden, dass die stationäre Behandlung der Schizophrenie mit einer geringeren Verbesserung des Funktionsniveaus einhergeht als bei anderen psychischen Störungen [33]. In Deutschland konnte eine prospektive Kohortenstudien von Patient:innen mit einer ersten psychotischen Störung eine Verringerung des psychopathologischen Schweregrads und einen Anstieg des Funktionsniveaus zeigen [32]. Ebenfalls konnten Studien bei Patient:innen mit längerer Krankheitsdauer Effekte der stationären Behandlung auf die Psychopathologie nachweisen [11, 38], auch wenn diese im Vergleich zu Ersterkrankten kleiner ausfielen [10]. Die Evaluation eines stationären Versorgungskonzepts für Patient:innen mit unterschiedlichen psychotischen Störungen sowie weitere aktuelle Studien zur Wirksamkeit der stationären Behandlung stehen hingegen noch aus.

In einem rezenten Artikel skizzieren Mehl und Kolleg:innen [23] leitliniengerechte stationäre Versorgung von Patient:innen mit Psychose anhand nationaler und internationaler Leitlinien. In ihrer Übersichtsarbeit werden zusätzlich zur medikamentösen Behandlung KVT, Psychoedukation, Training sozialer Kompetenzen, kognitives Training, Familieninterventionen sowie metakognitive Trainings besonders empfohlen. Darüber hinaus sollten Angehörige in die Therapie mit einbezogen werden und insbesondere Psychoedukation erhalten. Wir präsentieren die ersten Ergebnisse einer laufenden naturalistischen Studie zur stationären leitliniengerechten Behandlung von Patient:innen mit Psychosen. Neben Veränderungen in psychotischen Symptomen, im Funktionsniveau und in der Lebensqualität untersuchen wir auch Veränderungen in der Krankheitseinsicht, welche auch über die Übereinstimmung von Selbst- und Fremdratings erfasst werden kann [16]. Diese ist bei psychotischen Störungen häufig eingeschränkt und stellt einen bedeutsamen Prädiktor für die Adhärenz und das längerfristige Funktionsniveau dar [15].

## Methodik

In die vorliegende Untersuchung wurden Patient:innen der Forschungsstation für Psychosen am Zentrum für Psychiatrie Reichenau im Zeitraum von Dezember 2021 bis Mai 2025 eingeschlossen. Die Station fungiert als spezialisierte Diagnostik- und Therapiestation für Patient:innen mit psychotischen Störungen unterschiedlicher Ätiologie und in verschiedenen Krankheitsstadien mit offenem Stationssetting. Eine Aufnahme kann nach einer Stabilisierung auf einer Akutstation oder bei ausreichender Absprachefähigkeit direkt erfolgen. Das Behandlungskonzept für die postakute Phase orientiert sich an aktuellen Leitlinien, wie sie in Mehl et al. [23] dargestellt wurden: Zusätzlich zur psychopharmakologischen Behandlung werden Patient:innen von Ärzt:innen oder Psycholog:innen zweimal wöchentlich in Einzelgesprächen therapeutisch betreut und besuchen darüber hinaus – bei Indikation und Eignung – kognitive und metakognitive Trainings, ein Training sozialer Kompetenzen, eine psychoedukative Gesprächsgruppe sowie Sport- und Arbeitstherapie, eine Entspannungsgruppe und eine Abstinenzgruppe. Patient:innen werden aktiv in den Stationsalltag einbezogen und übernehmen alltägliche Dienste auf Station. Neben der fallbezogenen Einbeziehung von Angehörigen wird auch eine offene Angehörigengruppe angeboten. Die Studie wurde präregistriert (https://osf.io/v6p8h) und als klinische Studie registriert (https://www.drks.de/DRKS00028050).

Alle einwilligungsfähigen Patient:innen wurden nach der Aufnahme in Rücksprache mit dem Behandlungsteam über den geeigneten Zeitpunkt und bei ausreichender Deutschkenntnis vom Studienteam zur Studienteilnahme eingeladen. Die erste Untersuchung (T1) wurde zu Beginn des stationären Aufenthalts durchgeführt und möglichst kurz vor der Entlassung (T2) wiederholt. Beide Erhebungszeitpunkte beinhalteten Interviews zu Symptomatik und Funktionsniveau sowie Fragebögen und eine Aufgabe zur Entscheidungsfindung (Beads-Task). Für die vorliegende Arbeit sind die Interviews Positive and Negative Syndrom Scale (PANSS, [13]) und Personal and Social Performance Scale (PSP, [29]) relevant sowie die Fragebögen Psychotomimetic States Inventory (PSI, [20]), Brief Symptom Inventory (BSI, [5]) und WHOQOL-BREF [36]. Im Verlauf des Aufenthalts wurde außerdem die Diagnose mittels des SCID-Interviews nach DSM‑5 gesichert [2]. Antipsychotische Medikation wurden in Chlorpromazinäquivalente (CPZ) umgerechnet [3].

Nach Ausschluss von Patient:innen ohne aktuelle oder frühere Psychose, ermittelt anhand des SCID-Interviews, oder wegen wiederholter Studienteilnahme durch mehrmaligen Aufenthalt, lagen Daten von 96 Patient:innen zu T1 vor. Hiervon nahmen 31 nicht an T2 teil, sodass vollständige Daten von 65 Patient:innen zur Analyse zur Verfügung standen (Diagnosen siehe Zusatzmaterial, Stichprobencharakteristika siehe Tab. 1).

**Tab. 1:** Stichprobenmerkmale

*N* (weiblich, divers)	65 (20, 1)
Alter in Jahren (M, SD)	36,54 (10,34)
Schulabschluss (Abitur; Realschule; Hauptschule; ohne)	28; 18; 18; 1
Krankheitsdauer in Jahren (M, SD; Range; *n* = 60^a^)	13,86 (11,53; 0–39)
Aufenthaltsdauer auf der Station in Tagen (M, SD; Range)	60,42 (37,12; 21–239)
T1 Tage nach Aufnahme (M, SD)	8,48 (3,36)
T2 Tage bis Entlassung (M, SD)	2,46 (4,16)

*T1* Messung bei Aufnahme, *T2* Messung bei Entlassung

^a^Krankheitsdauer wurde durch Selbstauskunft erhoben, dabei gaben 5 Patient:innen an, niemals psychotische Symptome erlebt zu haben. Diese Patient:innen wurden von der Berechnung der Krankheitsdauer ausgeschlossen. Bei 4 dieser Patient:innen wurden Einschränkungen in der Krankheitseinsicht festgestellt (PANSS G12 zu T1 ≥ 3)

Die statistische Auswertung wurde mit *R* durchgeführt. Unterschiede in den untersuchten Skalen und Stichprobencharakteristika bei T1 zwischen der Stichprobe und ausgeschiedenen Patient:innen wurden mittels t‑Tests für unabhängige Stichproben und χ^2^-Tests oder bei Verletzung von Voraussetzungen mit Mann-Whitney-U-Tests untersucht. Drei Patient:innen der Dropout-Gruppe wurden aufgrund fehlender Daten von den Analysen ausgeschlossen.

Behandlungseffekte auf Symptomatik (PANSS: Gesamtwert und Subskalen Positivsymptome, Negativsymptome, Desorganisation und Affekt [31]; PSI: Summenwert aus den Subskalen Wahnhaftes Denken, Wahrnehmungsverzerrung und Paranoia; BSI: Global Severity Index (GSI)), Lebensqualität (WHOQOL-BREF-Globalwert) und Funktionsniveau (PSP) zwischen T1 und T2 wurden durch t‑Tests oder Wilcoxon-Signed-Rank-Tests (bei Verletzung von Annahmen) für abhängige Stichproben analysiert. Alle Maße erreichten eine interne Konsistenz von α > 0,70, außer die Skalen Desorganisation (α [T1] = 0,69) und Affekt des PANSS (α [T1] = 0,52, α [T2] = 0,38) sowie der Globalwerts des WHOQOL-BREF (α [T2] = 0,67). Zudem untersuchten wir ein klinisch bedeutsames Ansprechen auf die Behandlung (Response) als Verringerung des PANSS-Gesamtwertes um 25 %.

Veränderungen in der Krankheitseinsicht wurden zum einen anhand des PANSS-Items „Mangel an Urteilsfähigkeit und Einsicht“ (G12) untersucht. Zum anderen wurden zur Messung der Konkordanz von Selbst- und Fremdeinschätzung der Positivsymptomatik die entsprechenden Skalen aus PANSS (Positivsymptome) und PSI (Summenwert der Subskalen Wahnhaftes Denken, Wahrnehmungsverzerrung und Paranoia) korreliert. Um Änderungen in der Konkordanz zu untersuchen, verglichen wir die Korrelationsstärke zwischen den beiden Zeitpunkten mittels der Methode nach Zou implementiert im R‑Paket *cocor *[7].

## Ergebnisse

Die 65 Patient:innen hatten zu T1 im Vergleich zu den ausgeschiedenen Patient:innen einen signifikant geringeren Gesamtwert des PANSS (*W* = 1196,5, *p* = 0,017, *Mdn *Stichprobe = 53,0, *Mdn *Dropout = 59,5) sowie eine geringere Negativsymptomatik (*W* = 1161,5, *p* = 0,035, *Mdn *Stichprobe = 13,0, *Mdn* Dropout = 17,5). In den weiteren untersuchten Skalen und den Stichprobencharakteristika ergaben sich keine signifikanten Unterschiede.

**Abb. 1:**
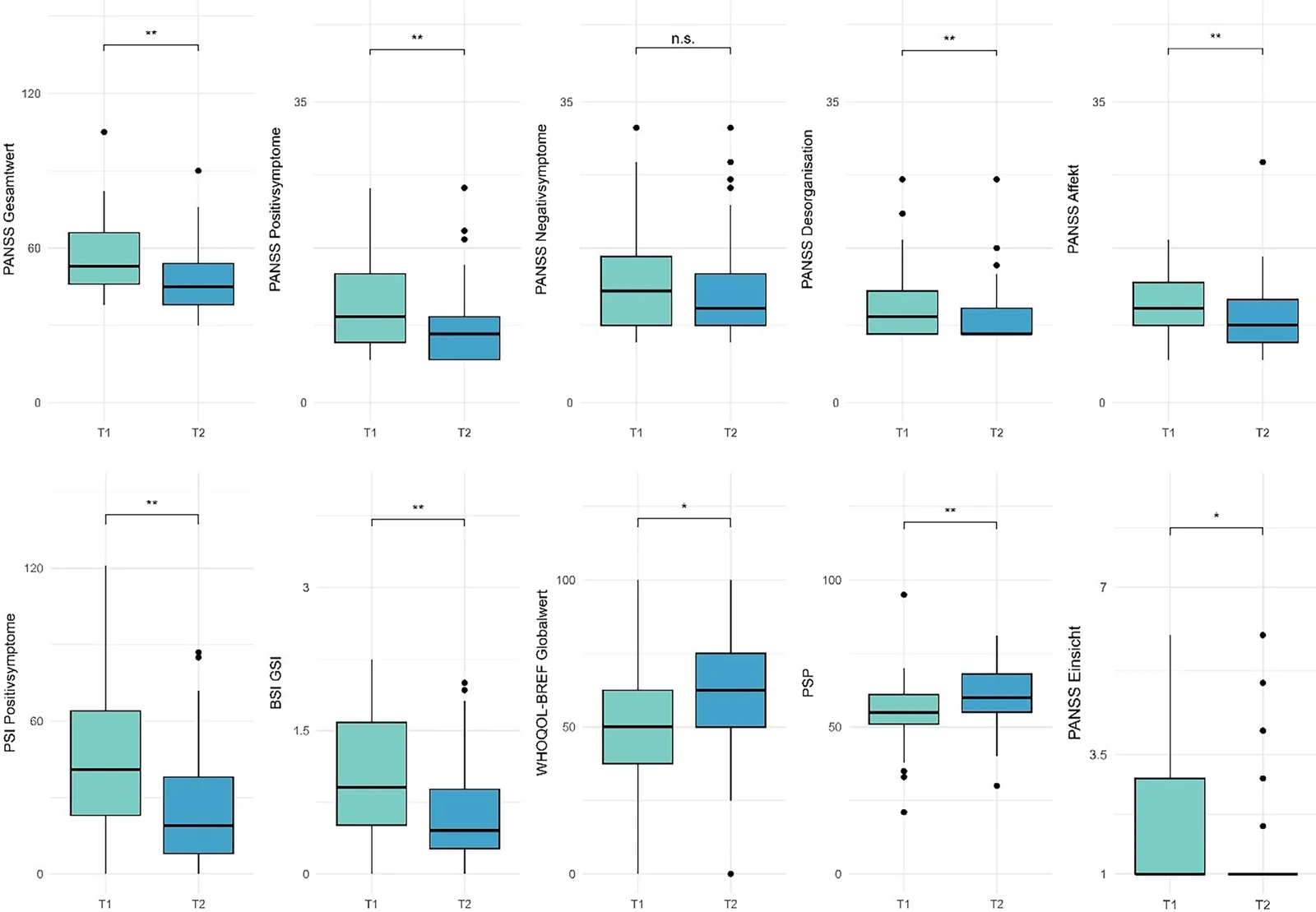
Veränderungen in Psychopathologie, Lebensqualität, Funktionsniveau und Krankheitseinsicht über den Verlauf der stationären Behandlung. Die Boxplots zeigen den Median als Mittellinie, die Außenlinien des Boxplots umfassen das 1. und 3. Quartil. Whisker entsprechen 1,5 * Interquartilsbereich, Punkte zeigen Werte außerhalb dieses Bereichs. PSI Positivsymptome wurde als Summenwert aus den Subskalen Wahnhaftes Denken, Wahrnehmungsverzerrung und Paranoia berechnet. *T1* Messung bei Aufnahme, *T2* Messung bei Entlassung, *PANSS* Positive and Negative Symptom Scale, *PSI* Psychotomimetic States Inventory, *BSI GSI* Global Severity Index des Brief Symptom Inventory, *PSP* Personal and Social Performance Scale, *n.* *s.* nicht signifikant, *p < 0,05, **p < 0,001

Die stationäre Behandlung führte zu einer signifikanten Verringerung im Gesamtwert des PANSS (*t [64]* = 7,87, *p* < 0,001, *d* = 0,98) sowie in den Subskalen Positivsymptome (*V* = 1156,5, *p* < 0,001, *r* = 0,62), Desorganisation (*V* = 803, *p* < 0,001, *r* = 0,45) und Affekt (*V* = 1408,5, *p* < 0,001, *r* = 0,67), aber nicht in der Negativsymptomatik (*t [64]* = 1,44, *p* = 0,154, *d* = 0,18). Auch die selbsteingeschätzte Positivsymptomatik im PSI verbesserte sich signifikant (*V* = 1681,5, *p* < 0,001, *r* = 0,57). Des Weiteren zeigte sich im BSI anhand des globalen Kennwerts GSI eine signifikante Symptomverringerung (*V* = 1913, *p* < 0,001, *r* = 0,73). Außerdem verbesserten sich die Lebensqualität, gemessen am Globalwert des WHOQOL-BREF (*V* = 234, *p* = 0,001, *r* = 0,41), sowie das Funktionsniveau im PSP (*V* = 216, *p* < 0,001, *r* = 0,56; Tab. 2 und Abb. 1)*.* Eine Response zeigte sich bei 43 (66 %) der Patient:innen.

**Tab. 2:** Psychopathologie und Medikation

	T1	T2
PANSS Gesamtwert	55,97 (13,09)	46,88 (11,83)
PANSS Positivsymptome	11,66 (5,56)	8,58 (4,44)
PANSS Negativsymptome	13,71 (5,55)	12,91 (5,77)
PANSS Desorganisation	11,54 (4,08)	9,98 (3,17)
PANSS Affekt	11,82 (3,5)	9,32 (3,95)
PSI Positivsymptome	45,45 (31,1)	25,82 (22,3)
BSI GSI	1,02 (0,64)	0,63 (0,51)
WHOQOL-BREF-Globalwert	51,54 (22,8)	59,62 (20,1)
PSP	56,12 (11,04)	61,17 (10,49)
PANSS Einsicht (G12)	1,88 (1,41)	1,49 (1,13)
CPZ-Äquivalent	610,2 (325,88; 0–1312,5)	588,2 (371,88; 0–1500)

PANSS Skalenzuordnung nach [31]. PSI Positivsymptome wurde als Summenwert aus den Subskalen Wahnhaftes Denken, Wahrnehmungsverzerrung und Paranoia berechnet

*T1* Messung bei Aufnahme, *T2* Messung bei Entlassung, *CPZ* Chlorpromazin

Außerdem hatte die Behandlung eine Verbesserung in der fremdeingeschätzten Krankheitseinsicht, gemessen anhand des Items G12 des PANSS, zur Folge (*V* = 229, *p* = 0,021, *r* = 0,29, Abb. 1). Die selbsteingeschätzte Positivsymptomatik korrelierte signifikant mit der fremdeingeschätzten Positivsymptomatik zu beiden Messzeitpunkten (T1 *r*_*s*_ = 0,557, *p* < 0,001; T2 *r*_*s*_ = 0,558, *p* < 0,001). Die Korrelationsstärke veränderte sich über den Verlauf der Behandlung nicht signifikant (95 %-KI [−0,24 0,24]).

## Diskussion

Unsere naturalistische Verlaufsstudie auf einer spezialisierten Therapiestation für Patient:innen mit Psychosen zeigt signifikante Effekte auf Gesamtsymptomatik, Positivsymptomatik, Desorganisation und affektive Symptome. Ein klinisch bedeutsames Ansprechen auf die Behandlung beobachteten wir bei 66 % der Patient:innen. Die Negativsymptomatik hingegen schien resistent. Darüber hinaus ergab sich eine signifikante Verbesserung der Lebensqualität und des Funktionsniveaus der Patient:innen. Die Therapie zeigte sich effektiv in der Verbesserung der fremdeingeschätzten Krankheitseinsicht der Patient:innen. Die Ergebnisse lassen auf eine hohe Konkordanz von Fremd- und Selbsteinschätzung schließen, die sich allerdings im Verlauf der Behandlung nicht verbesserte.

Unsere Befunde decken sich mit rezenten Metaanalysen von RCTs zu stationärer Therapie mit psychologischen Interventionen, in denen positive Effekte für Positivsymptomatik, Lebensqualität und Funktionsniveau der Patient:innen gefunden wurden [1, 37]. Die relative Behandlungsresistenz der Negativsymptomatik im Rahmen der stationären Therapie in unserer Stichprobe könnte auf die Behandlungszeit von im Mittel 60 Tagen zurückzuführen sein. In Übereinstimmung damit berichtete eine Metaanalyse, dass sich signifikante Verbesserungen der Negativsymptomatik bei psychologischen Interventionen im akuten stationären Setting erst im Katamnesezeitraum, nicht aber direkt nach der Intervention entfalteten [37]. Negativsymptome verändern sich in der Regel über längere Zeiträume [28] und Metaanalysen zu ambulanten Therapien finden meist signifikante Verbesserungen [17]. Dies unterstreicht die Wichtigkeit einer längerfristigen psychotherapeutischen Begleitung, auch außerhalb der stationären Behandlung bei Patient:innen mit Psychosen [cf. 30]. Zudem zeigen Studien zu adjunktiven Verfahren, wie Sportinterventionen bei längerer Dauer [27] und spezifische achtsamkeits- und akzeptanzbasierte Verfahren [12], vielversprechende Ergebnisse zur Reduktion von Negativsymptomatik. Hier könnten Ansatzpunkte für eine Verbesserung der Wirksamkeit der stationären Therapie liegen.

Im Einklang mit bisherigen Befunden beobachteten wir außerdem eine Verbesserung der fremdeingeschätzten Krankheitseinsicht [25]. Die Konkordanz von Selbst- und Fremdeinschätzung der Positivsymptomatik veränderte sich hingegen nicht und war bereits zu Behandlungsbeginn gegeben. Dieses Ergebnis deckt sich mit Befunden, dass die Einschätzungen der Symptomatik erst ab einem hohen Einschränkungsgrad der Krankheitseinsicht divergieren [18] und generell eine gute Übereinstimmung zu erwarten ist (z. B. [16, 19]). Selbstauskunft scheint also auch bei Psychosepatient:innen eine zuverlässige Grundlage zur Einschätzung der Psychopathologie darzustellen [cf. 18]. Zukünftige Studien könnten daher bei der Evaluation der Effektivität stationärer Therapie in dieser Patient:innengruppe stärker auf Fragebogenmaße zurückgreifen.

Unsere Studie hat einige Limitationen. Zum einen handelt es sich um eine naturalistische Verlaufsstudie, die somit weder eine Standardisierung der Intervention noch eine Kontrollgruppe und randomisierte Zuordnung beinhaltet. Der Vergleich zu frühen Befunden unbehandelter Krankheitsverläufe legt allerdings die Effektivität der stationären Behandlung nahe [26]. Darüber hinaus wurden alle Interviews ohne Verblindung gegenüber dem Untersuchungszeitpunkt durchgeführt, was zu einer Überschätzung des Therapieerfolgs führen könnte. Dagegen spricht allerdings die gute Übereinstimmung zwischen Fremd- und Selbsteinschätzung, die sich nicht zwischen den Erhebungszeitpunkten unterschied. Außerdem zeigten die Skalen Desorganisation und Affekt des PANSS sowie der WHOQOL-BREF-Globalwert eine niedrige interne Konsistenz, wodurch die Interpretierbarkeit der Skalen beeinträchtigt sein kann. Abschließend handelt es sich um eine relativ kleine Stichprobe und Patient:innen, welche die Studienteilnahme abbrachen, waren von einer höherer Gesamt- und Negativsymptomatik charakterisiert. Hierdurch kann die externe Validität der Ergebnisse eingeschränkt sein. Die Studie wird fortlaufend erhoben, sodass wir in einigen Jahren aktualisierte Ergebnisse präsentieren und die Daten auch zur Vorhersage von Therapieverläufen nutzen können.

Zusammenfassend legen die Ergebnisse nahe, dass stationäre Therapie für Patient:innen mit Psychosen nach aktuellen Leitlinien zur Behandlung der Schizophrenie effektiv in der Reduktion der Symptomatik und in der Verbesserung von Einsicht, Lebensqualität und Funktionsniveau ist. Unsere Ergebnisse ergänzen bisherige Erkenntnisse zur Wirksamkeit stationärer Therapie um realitätsnahe Daten aus der Praxis. Wir hoffen, dass diese „practice-based evidence“ [4] klinisch tätige Kolleg:innen in ihrer Arbeit bestätigt und darin ermutigt, Patient:innen mit Psychosen eine leitliniengerechte Behandlung mit umfassendem psychotherapeutischem Programm anzubieten und zur Erforschung der Effektivität stationärer Therapien beizutragen.

## Fazit für die Praxis

- Leitliniengerechte stationäre Therapie ist effektiv in der Verringerung der Symptomatik sowie in der Verbesserung des Funktionsniveaus, der Lebensqualität und der Krankheitseinsicht von Patient:innen mit Psychosen.
- Negativsymptomatik wird im Rahmen der Dauer einer stationären Behandlung nur wenig beeinflusst, sodass eine längerfristige ambulante Therapie angestrebt werden sollte.
- Patient:innen mit Psychosen können ihre eigene Positivsymptomatik einschätzen. Fragebögen zur psychotischen Symptomatik können daher unterstützend zur Evaluation des Therapieerfolges genutzt werden.

## Supplementary Information

eTab. 1 Häufigkeit der Diagnosen nach DSM‑5

## Data Availability

Die Daten wurden im Rahmen einer laufenden Studie erhoben. Eine Veröffentlichung ist zukünftig unter Berücksichtigung der datenschutzrechtlichen Bestimmungen geplant und wird in der klinischen Registrierung (https://www.drks.de/DRKS00028050) bekannt gegeben.
