# Supplementary material for: The effectiveness of inpatient treatment of psychoses—Initial results of a naturalistic study
Source: Nervenarzt. 2025 Aug 19;97(4):363–9. [Article in German] doi: 10.1007/s00115-025-01871-1 (PMC13314691; doi:10.1007/s00115-025-01871-1)
Supplement: Supplementary file 1 — eTab. 1 Häufigkeit der Diagnosen nach DSM‑5 [file 115_2025_1871_MOESM1_ESM.docx]

# Zusatzmaterial

# Die Effektivität stationärer Behandlung von Psychosen – erste Ergebnisse einer naturalistischen Studie

**Tab. 1** *Häufigkeit der Diagnosen nach DSM-5*

|  | Hauptdiagnose | Nebendiagnose |
| --- | --- | --- |
| Schizophrenie | 36 | 2 |
| Schizoaffektive Störung | 5 | - |
| Schizophrenieforme Störung | 1 | - |
| Wahnhafte Störung | 2 | - |
| Kurze Psychotische Störung | 1 | 1 |
| Andere Schizophrenie-Spektrum und andere psychotische Störung | 2 | - |
| Substanz- /Medikamenteninduzierte Psychotische Störung | 5 | 1 |
| Schizotype Persönlichkeitsstörung | 1 | 1 |
| Bipolare Störung | 6 | 1 |
| Major Depression | 2 | 14 |
| Panikstörung | 1 | - |
| Schizoide Persönlichkeitsstörung | 1 | 1 |
| Borderline Persönlichkeitsstörung | 1 | 1 |
| Aufmerksamkeitsdefizit-/Hyperaktivitätsstörung | 1 | 1 |
| Störung durch Substanzkonsum | - | 19 |
| Agoraphobie | - | 3 |
| Soziale Angststörung | - | 2 |
| Spezifische Phobie | - | 2 |
| Generalisierte Angststörung | - | 2 |
| Posttraumatische Belastungsstörung | - | 1 |
| Bulimia Nervosa | - | 1 |
| Paranoide Persönlichkeitsstörung | - | 1 |
| Antisoziale Persönlichkeitsstörung | - | 1 |
